# Association between microscopic brain damage as indicated by magnetization transfer imaging and anticardiolipin antibodies in neuropsychiatric lupus

**DOI:** 10.1186/ar1892

**Published:** 2006-01-16

**Authors:** Stefan CA Steens, Gerlof PTh Bosma, Gerda M Steup-Beekman, Saskia le Cessie, Tom WJ Huizinga, Mark A van Buchem

**Affiliations:** 1 Department of Radiology, Leiden University Medical Center, Leiden, The Netherlands; 2 Department of Rheumatology, Leiden University Medical Center, Leiden, The Netherlands; 3 Department of Medical Statistics and Bio-informatics, Leiden University Medical Center, Leiden, The Netherlands

## Abstract

The pathogenetic role of anticardiolipin antibodies (aCLs) in patients with neuropsychiatric systemic lupus erythematosus (NPSLE) without cerebral infarcts remains elusive. Magnetization transfer imaging (MTI) has proved to be a sensitive tool for detecting diffuse microscopic brain damage in NPSLE patients. In this study we examined the correlation between grey and white matter magnetization transfer ratio (MTR) parameters and the presence of IgM and IgG aCLs and lupus anticoagulant in 18 patients with systemic lupus erythematosus and a history of NPSLE but without cerebral infarcts on conventional magnetic resonance imaging. Lower grey matter mean MTR (*P *< 0.05), white matter mean MTR (*P *< 0.05), white matter peak location (*P *< 0.05) and grey matter peak location (trend toward statistical significance) were observed in IgM aCL-positive patients than in IgM aCL-negative patients. No significant differences were found in MTR histogram parameters with respect to IgG aCL and lupus anticoagulant status, nor with respect to anti-dsDNA or anti-ENA (extractable nuclear antigen) status. This is the first report of an association between the presence of aCLs and cerebral damage in grey and white matter in NPSLE. Our findings suggest that aCLs are associated with diffuse brain involvement in NPSLE patients.

## Introduction

Central nervous system (CNS) involvement causes neuropsychiatric manifestations in up to 75% of patients with systemic lupus erythematosus (SLE) [1]. If these neuropsychiatric symptoms are not attributable to secondary factors such as infections, medication, or metabolic derangements, then they can often be attributed to the SLE disease directly affecting the CNS [2,3]. In SLE patients with neuropsychiatric manifestations such as cognitive dysfunction, conventional magnetic resonance imaging (MRI) may be unremarkable or show only nonspecific abnormalities [4]. Nevertheless, using magnetization transfer imaging (MTI) – a quantitative MRI technique that is sensitive to macroscopic and microscopic brain tissue changes [5] – global brain involvement has been detected in patients with neuropsychiatric systemic lupus erythematosus (NPSLE) without explanatory abnormalities on conventional MRI [6-8]. Correlations have been reported between MTI parameters and measures of neurologic, psychiatric and cognitive function [9], as well as parameters from other quantitative neuroimaging techniques [10].

The pathogenesis of neuropsychiatric symptoms in SLE patients without explanatory MRI abnormalities remains largely unknown [3]. Various autoantibodies have been implicated in the pathogenesis of NPSLE, including anticardiolipin antibodies (aCLs) [11,12]. Because of their prothrombotic tendency, aCLs may cause cerebral infarctions and as such they are correlated with focal neurological syndromes [13-15]. Although associations with nonfocal neuropsychiatric manifestations have been reported [16-20], the role of aCLs in the pathogenesis of neuropsychiatric symptoms in patients without cerebral infarcts is less clear. The aim of the present study was to evaluate whether the presence of aCLs in SLE patients with a history of neuropsychiatric manifestations but without explanatory abnormalities on conventional MRI is associated with brain involvement detected by MTI.

## Materials and methods

### Study design

In this study we examined the relation between brain damage as indicated by quantitative MTI parameters and the presence of aCLs, lupus anticoagulant (Lac) and antibodies directed against DNA and extractable nuclear antigen (ENA).

### Participants

Eighteen female patients diagnosed with SLE in accordance with the 1982 revised American College of Rheumatology (ACR) criteria [21] and with a history of CNS involvement were asked to participate (age 23–65 years, mean 34 years). The mean SLE disease duration was nine years (range 7 months to 29 years); neuropsychiatric symptoms had been diagnosed one month to 18 years (mean 5 years) before scanning. At the time of the study, no active neuropsychiatric symptoms or any concurrent other neurological or psychiatric diseases were present. Patients with radiological evidence of cerebral infarctions were not included. Before laboratory and imaging data were acquired, all patients were classified according to the 1999 ACR NPSLE case definitions [2] by one experienced rheumatologist. None of the patients had clinical symptoms compatible with the antiphosphlipid syndrome. The institutional review board approved the research protocol, and informed consent was obtained.

### Laboratory examination

Mean time between the MRI/MTI examination and laboratory examination was 1.3 days (range 0–13 days). The presence of IgM and IgG aCLs (phospholipid units/ml) was assessed using commercial ELISA kits (Pharmacia & Upjohn Diagnostics GmbH, Freiburg, Germany) in a procedure that is standard in our rheumatology department. The assays used for the detection of Lac were lupus-aPTT (activated partial thromboplastin time) and LA-screen and LA-confirm (Gradipore Inc, New York, NY, USA). The presence of antibodies against ENA (anti-ENA) was assessed using QUANTA Lite™ ENA 6 ELISA kit (INOVA Diagnostics Inc, San Diego, CA, USA); an immunofluorescent assay (Biomedical Diagnostics, Antwerp, Belgium) was used to detect antibodies against double-stranded DNA (anti-dsDNA).

### Magnetic resonance imaging protocol

MRI was carried out on a Philips Gyroscan Intera ACS-NT 1.5 T MR scanner (Philips Medical Systems, Best, The Netherlands). Scans were aligned parallel to the axial plane through the anterior and posterior commissure and covered the whole brain in all sequences. Conventional T1-weighted spin-echo, fluid-attenuated inversion recovery and dual (fast spin-echo proton density and T2-weighted) images were acquired in all patients and interpreted by one experienced neuroradiologist [9]. Subsequently, MTI was performed using a three-dimensional gradient-echo pulse sequence with a TE (echo time) of 6 ms, TR (repetition time) of 106 ms and a flip angle of 12°. Scan parameters were chosen to minimize T1 and T2 weighting, resulting in proton density contrast in the absence of magnetization transfer saturation pulses [22]. A matrix of 128 × 256 pixels was used for 28 contiguous slices, with 5 mm slice thickness and a field of view of 220 mm. Two consecutive sets of axial images were acquired: the first with and the second without a sinc-shaped radio frequency saturation pulse 1,100 Hz upfield of H_2_O resonance. Scanning time for MTI was 11 min and 21 s [23].

### Image processing

All analyses were performed by one observer. Using the software platform SNIPER (Software for Neuro-Image Processing in Experimental Research; Division of Image Processing, Department of Radiology, Leiden University Medical Center, Leiden, The Netherlands) on an offline workstation, the magnetization transfer ratio (MTR) was calculated per voxel using the equation MTR = ([M_0 _- M_s_])/M_0_) × 100%, with M_0 _and M_s _representing the intensity of voxels in a nonsaturated state and in a saturated state, respectively [5]. Then, MTR histograms were generated for grey matter and white matter separately according to a method described previously [23] using Statistical Parametric Mapping '99 (Wellcome Department of Cognitive Neurology, Institute of Neurology, London, UK [24]). In summary, M_s _images were segmented and probability maps for grey matter, white matter and cerebrospinal fluid were produced automatically. All images were inspected visually to confirm adequate extraction of intracranial contents. Binary masks were then produced for grey matter and white matter separately based on conservative thresholds to avoid partial voluming at tissue interfaces, and these binary masks were applied to the original MTR maps producing grey matter and white matter MTR maps. From these MTR maps, grey matter and white matter MTR histograms were generated and normalized for volume differences. Then, the mean MTR (percent unit), peak height (arbitrary unit) and peak location (percent unit) were read from the normalized histogram without any function fitting [23]. The mean MTR indicates the average MTR value, the peak height is a measure of the uniformity of brain tissue in terms of MTR values, and the peak location is an indicator of the MTR value occurring most often. In NPSLE, lowering of MTR values probably indicates neuronal and axonal injury, atrophy, or demyelination or gliosis [10].

### Statistical analysis

Average and standard deviation were calculated for the clinical parameters age, duration of SLE and duration of NPSLE, and for the grey and white matter MTR histogram parameters mean MTR, peak height and peak location. Nonparametric Mann–Whitney tests were performed to compare clinical and grey and white matter MTR histogram parameters between patients with and without IgM aCLs, IgG aCLs, Lac, anti-dsDNA and anti-ENA (SPSS for Windows, Rel. 11, 2002; SPSS Inc., Chicago. IL, USA).

## Results

Table 1 lists the observed NPSLE manifestations according to 1999 ACR case definitions [2], antibody status, and findings on conventional MRI. Nine patients tested positive for IgM aCLs, nine for IgG aCLs and 13 for Lac, yielding a comparison of nine versus nine patients for IgM aCL, nine versus nine patients for IgG aCL, and 13 versus five patients for Lac. For anti-dsDNA and anti-ENA, four and 11 patients tested positive, respectively. In two patients, anti-dsDNA or anti-ENA status was unavailable.

**Figure 1:**
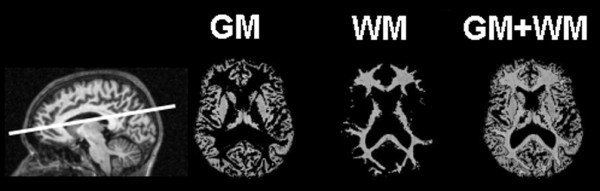
Example of segmented axial MTR map (level indicated at the sagittal image). Visualized are the compartments grey matter (GM), white matter (WM) and grey and white matter (GM + WM). Signal intensities represent MTR values. MTR, magnetization transfer ratio.

**Figure 2:**
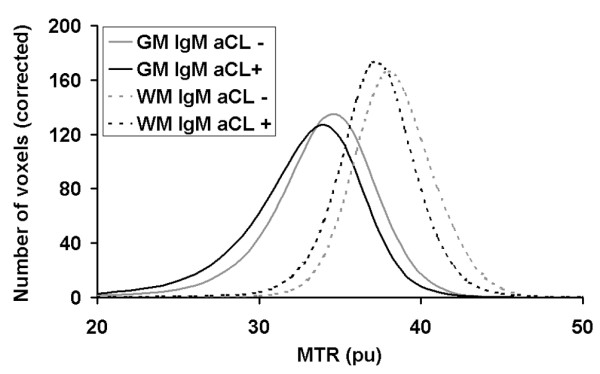
Average MTR histograms after volume corrections for patients with and without IgM aCLs. Visualized are the average MTR histograms for patients with IgM aCLs (black lines) and patients without IgM aCLs (grey lines) for the grey matter (GM; continous lines) and white matter (WM; dashed lines). aCL, anticardiolipin antibodies; MTR, magnetization transfer ratio.

All images showed accurate segmentation of grey and white matter, an example of which is shown in Figure 1. The stringent probability thresholds excluded voxels with a partial volume effect at the interfaces of grey matter, white matter and cerebrospinal fluid, providing pure grey and white matter MTR maps (Figure 1). Grey and white matter MTR values showed considerable overlap, with higher MTR values for the white matter (Figure 2).

**Figure 3:**
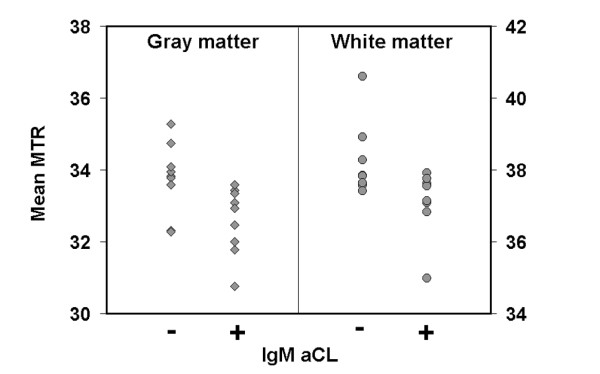
Plot of the mean of the MTR histogram for patients with and without IgM aCLs. Visualized are the mean MTRs for patients with IgM aCLs versus patients without IgM aCLs for the grey matter (GM) and white matter (WM). aCL, anticardiolipin antibodies; MTR, magnetization transfer ratio.

Mann–Whitney tests revealed a lower grey matter mean MTR, white matter mean MTR and white matter peak location (*P *< 0.05) and grey matter peak location (trend toward significance) in IgM aCL-positive as compared with IgM aCL-negative patients (Table 2, Figure 3). Lower values were also observed for grey and white matter mean MTR and peak location in IgG aCL-positive than in IgG aCL-negative patients (not significant) and in Lac-positive than in Lac-negative patients (trend toward significance for grey and white matter mean MTR). No significant differences were found for the MTR histogram parameters with respect to anti-dsDNA or anti-ENA status (*P *> 0.2 and *P *> 0.3 for all MTR parameters, respectively), or for age or SLE or NPSLE disease duration in all comparisons.

## Discussion

This is the first study to investigate the relation between MTI parameters of the brain and aCLs in NPSLE patients. MTI parameters demonstrated brain damage in aCL-positive SLE patients in the absence of explanatory abnormalities on conventional MRI. Therefore, our results suggest that, apart from giving rise to macroscopic cerebral infarctions, aCLs may play a role in the pathogenesis of diffuse microscopic brain damage in NPSLE.

MTI has proved to be a sensitive tool for detecting diffuse brain involvement in NPSLE patients [4]. In previous work, based on whole-brain MTR histograms, it was found that SLE patients with active neuropsychiatric symptoms, past neuropsychiatric symptoms, and SLE patients without neuropsychiatric symptoms could be distinguished, suggesting diagnostic potential for these parameters [6-8]. The previously observed correlations between whole-brain MTR histogram parameters and measures of neurological, psychiatric and cognitive function [9] emphasized the functional relevance of MTI parameters in such patients. In the present study SLE patients with a history of neuropsychiatric symptoms were included. Apart from overt diffuse neuropsychiatric manifestations, some patients suffered from chronic multifocal neuropsychiatric symptoms and were classified as having cerebrovascular disease, subclassification chronic multifocal disease [2]. Although two of the four patients classified as such exhibited nonspecific MRI abnormalities, in none of the patients was there evidence of cerebral infarcts or any other abnormality on conventional MRI to explain their neuropsychiatric symptoms. Therefore, in all patients diffuse involvement of the CNS was thought to underlie the neuropsychiatric manifestations. We observed lower values for mean MTR and peak location in grey and white matter in patients positive for aCLs and Lac.

The pathological conditions underlying the MTR histogram abnormalities and neuropsychiatric manifestations in SLE patients remain unclear. Although neuropathological studies in NPSLE patients are limited, vasculopathy and microinfarcts have been noted in several studies [3]. A recent MTI study examining cerebral grey and white matter separately in SLE patients with a history of diffuse neuropsychiatric manifestations [23] identified MTR histogram abnormalities specifically in the grey matter, suggesting that neuronal injury is among the key factors in diffuse NPSLE. This hypothesis is supported by increased levels of neuronal and astrocytic degradation products observed in the cerebrospinal fluid of NPSLE patients [25]. Microscopic brain damage was also suggested given the data from other quantitative neuroimaging techniques, such as magnetic resonance spectroscopy [26-31], spin-spin relaxation time measurements [32] and diffusion-weighted imaging [33]. A recent study combining these MRI techniques [10] indicated that the presence of neuronal and axonal injury, atrophy, demyelination and gliosis are aspects of the processes involved in neuropsychiatric involvement in SLE.

Although several studies have reported abnormalities on conventional MRI in patients with antiphospholipid antibodies [34-36], to our knowledge the only previous MTI study in patients with a known antiphospholipid antibody status is that by Rovaris and coworkers [8]. That study included healthy control individuals, patients suffering from SLE with and without neuropsychiatric symptoms, and patients suffering from the antiphospholipid antibody syndrome. No significant differences were observed between the patients with antiphospholipid antibody syndrome patients and healthy control individuals, whereas lower mean MTR values were observed in NPSLE patients than in non-NPSLE patients. These observations and the findings of our study suggest that the mere presence of antiphospholipid antibodies, including aCLs, does not lead to diffuse microscopic brain damage as detected by MTI, but they implicate that aCLs are involved in the pathogenetic events that lead to neuropsychiatric manifestations in SLE. A role for antiphospholipid antibodies in the pathogenesis of NPSLE has also been suggested by studies using magnetic resonance spectroscopy. In a study conducted by Sabet and coworkers [28], a reduced *N*-acetyl-aspartate to creatine ratio suggesting neuronal loss or injury was observed in SLE patients with the antiphospholipid antibody syndrome, as compared with SLE patients without – an effect that was mainly attributed to the presence of IgG aCLs.

Much in the order of the pathogenetic events that occur in SLE patients with diffuse neuropsychiatric manifestations remains unknown, although evidence for involvement of antineuronal antibodies, complement activation and proinflammatory cytokines has been found [3]. There are at least three possible explanations for how aCLs could be involved. First, the thrombotic tendency of antiphospholipid antibodies, including aCLs, may cause aggregation of thrombocytes and an increase in blood viscosity [3,11,37]. This may affect blood flow in small cerebral blood vessels in particular and cause widespread hypoperfusion, which subsequently causes ischaemic damage to brain tissue [38]. The trend observed with Lac in the present study supports this hypothesis. Second, aCLs may activate endothelial cells and cause a diffuse small-vessel vasculopathy – a neuropathological finding that was reported as long ago as 1968 [3,11,37-39]. The resulting increase in blood–brain barrier permeability permits entrance to the brain parenchyma of substances such as circulating antibodies [3,40]. Third, it has been shown *in vitro *that IgG aCLs themselves may interfere with glutamatergic pathways by a mechanism involving over-activation of the *N*-methyl-d-aspartate receptor [41,42].

The present study has several limitiations, and the results are preliminary. First, patient numbers were small, and control individuals were not available. Second, aCL status at the time of active neuropsychiatric manifestations was not available in this SLE patient cohort with past neuropsychiatric symptoms, which precludes evaluation of our results in the light of fluctuation in aCL levels [19]. Possibly, an even stronger association could be found between MTI measures of brain damage and aCL status at the time of active neuropsychiatric symptomatology. A prospective study should therefore include a larger NPSLE patient group with inactive and active neuropsychiatric symptoms, as well as control groups consisting of non-NPSLE patients and patients suffering from similar neuropsychiatric conditions, preferably with measurements of aCLs in serum and cerebrospinal fluid. Also, the specific role of IgM and IgG aCLs remains to be identified.

## Conclusion

This is the first study to find an association between aCLs and brain damage as detected by MTI in NPSLE patients. These results suggest that aCLs, in addition to contributing to overt brain infarcts, may also contribute to widespread microscopic damage in the brain.

## Abbreviations

aCL = anticardiolipin antibody; ACR = American College of Rheumatology; CNS = central nervous system; ELISA = enzyme-linked immunosorbent assay; ENA = extractable nuclear antigen; Lac = lupus anticoagulant; MRI = magnetic resonance imaging; MTI = magnetization transfer imaging; MTR = magnetization transfer ratio; NPSLE = neuropsychiatric systemic lupus erythematosus; SLE = systemic lupus erythematosus;

## Competing interests

The authors declare that they have no competing interests.

## Authors' contributions

SCAS,GPTB,TWJH and MAvB participated in the design of the study. SCAS, GPTB and GMS performed a literature search. SCAS, GPTB and GMS carried out data acquisition. SCAS, GMS, TWJH and MAvB carried out data analysis. SCAS and SleC performed the statistical analysis. SCAS, GPTB, GMS, SleC, TWJH and MAvB drafted the manuscript. All authors read and approved the final manuscript.

## Figures and Tables

**Table 1:** Patient characteristics, NPSLE manifestations and abnormalities detected on conventional MRI

Age (years)	Neuropsychiatric symptoms^a^	IgM aCLs	IgG aCLs	Lac	Anti-dsDNA	Anti-ENA	Radiological abnormalities
23	Acute confusional state	-	-	-	+	+	NDA
24	Primary generalized tonic clonic seizures	+	+	+	-	-	NDA
25	Primary generalized absence seizures	+	+	+	-	-	PAIS (6 lesions, 4 mm) cerebral atrophy
26	Primary generalized absence seizures	-	+	-	-	+	NDA
27	Primary generalized tonic clonic seizures	+	+	+	+	+	NDA
27	Primary generalized tonic clonic seizures	-	-	+	-	+	NDA
29	Cerebrovascular disease^b^	+	-	+	-	NA	NDA
30	Anxiety disorder	-	-	-	-	+	NDA
30	Cognitive dysfunction	-	+	+	-	+	NDA
30	Aseptic meningitis	-	-	-	-	+	NDA
32	Cerebrovascular disease^b^	+	+	+	NA	-	PAIS (29 lesions, 6 mm) cerebral atrophy, cerebellar infarction (9 mm)
36	Cerebrovascular disease^b^; cognitive dysfunction	-	-	+	-	-	NDA
38	Primary generalized tonic clonic seizures	+	+	+	-	-	PAIS (2 lesions, 6 mm) cerebral atrophy, cerebellar infarction (9 mm)
39	Aseptic meningitis	+	+	+	-	+	NDA
41	Chorea	-	-	+	+	+	PAIS (7 lesions, 4 mm)
41	Mononeuropathy (single); cognitive dysfunction	-	+	+	+	+	PAIS (1 lesion, 7 mm)
49	Mood disorder with depressive features	+	-	-	-	-	PAIS (21 lesions, 3 mm)
65	Cerebrovascular disease^b^	+	-	+	-	+	CAIS

^a^According to the American College of Rheumatology (ACR) nomenclature and case definitions for neuropsychiatric lupus syndromes [2]. ^b^Chronic multifocal disease. aCL, anticardiolipin antibody; anti-dsDNA, antibodies directed agains double stranded DNA; anti-ENA, antibodies directed against extractable nuclear antigen; Lac, lupus anticoagulant; MRI, magnetic resonance imaging; NA, not available; NDA, no detectable abnormalities on conventional MRI; CAIS/PAIS, confluent and punctate areas of increased signal (number of lesions, mean size of lesions); NPSLE, neuropsychiatric systemic lupus erythematosus.

**Table 2:** Descriptive statistics and Mann–Whitney test results

Parameter	IgM aCL^+^	IgM aCL^-^	*P*	IgG aCL^+^	IgG aCL^-^	*P*	Lac^+^	Lac^-^	*P*
Number of patients	9	9	-	9	9	-	13	5	-
Age (years)	36.4 ± 13.4	31.6 ± 6.4	0.67	31.3 ± 6.5	36.7 ± 13.2	0.49	34.9 ± 10.9	31.6 ± 10.2	0.50
Duration of SLE (years)	7.4 ± 5.0	10.6 ± 8.8	0.55	8.4 ± 3.6	9.6 ± 9.8	0.67	8.8 ± 7.1	9.6 ± 8.0	0.85
Duration of NPSLE (years)	4.4 ± 4.0	6.2 ± 5.5	0.49	4.9 ± 3.2	5.7 ± 6.1	0.73	5.7 ± 5.2	4.3 ± 3.7	0.57
Grey matter peak location	33.8 ± 0.7	34.7 ± 1.0	0.077	34.1 ± 0.3	34.3 ± 1.3	0.67	34.0 ± 0.1	34.8 ± 1.3	0.34
Grey matter peak height	131 ± 24	138 ± 19	0.67	135 ± 28	133 ± 14	0.93	133 ± 22	138 ± 23	0.63
Grey matter mean MTR	32.6 ± 0.9	33.8 ± 1.0	0.011	33.0 ± 0.8	33.3 ± 1.4	0.49	32.9 ± 1.1	33.8 ± 1.1	0.12
White matter peak location	37.2 ± 1.0	38.4 ± 1.0	0.019	37.8 ± 0.4	37.9 ± 1.6	0.93	37.6 ± 1.0	38.4 ± 1.6	0.50
White matter peak height	184 ± 31	178 ± 20	0.26	185 ± 32	177 ± 18	0.16	180 ± 27	183 ± 24	0.99
White matter mean MTR	37.2 ± 0.9	38.2 ± 1.0	0.014	37.6 ± 0.3	37.8 ± 1.5	0.44	37.4 ± 0.9	38.4 ± 1.3	0.14

Listed are the mean values ± standard deviation for IgM-positive/IgM-negative and IgG-positive/IgG-negative aCLs as well as Lac, and *P *values of Mann–Whitney tests between the groups. aCL, anticardiolipin antibody; Lac, lupus anticoagulant; MTR, magnetization transfer ratio; NPSLE, neuropsychiatric systemic lupus erythematosus; SLE, systemic lupus erythematosus.
